# Genetic Characterization and Molecular Evolution of Urban Seoul Virus in Southern China

**DOI:** 10.3390/v11121137

**Published:** 2019-12-09

**Authors:** Qianqian Su, Yi Chen, Meng Li, Jiajun Ma, Bo Wang, Jing Luo, Hongxuan He

**Affiliations:** 1 National Research Center for Wildlife-Borne Diseases, Institute of Zoology, Chinese Academy of Sciences, Beijing 100101, China; suqianqian@ioz.ac.cn (Q.S.); limeng@ioz.ac.cn (M.L.); majiajun93@126.com (J.M.); wangbo@ioz.ac.cn (B.W.); luojing@ioz.ac.cn (J.L.); 2 University of Chinese Academy of Sciences, Beijing 100101, China; chenyi@ioz.ac.cn; 3 State Key Laboratory of Integrated Management of Pest Insects and Rodents in Agriculture, Institute of Zoology, Chinese Academy of Sciences, Beijing 100101, China

**Keywords:** evolution, purifying selection, recombination, *Rattus norvegicus*, Seoul virus, substitution rate, urbanization, mutational saturation

## Abstract

Seoul virus (SEOV), which causes hemorrhagic fever with renal syndrome (HFRS) in humans, has spread all over the world, especially in mainland China. Understanding basic mechanisms of SEOV evolution is essential to better combat and prevent viral diseases. Here, we examined SEOV prevalence and evolution in the residential area of four districts in Guangzhou city, China. The carriage of SEOV was observed in 33.33% of the sampled rodents, with 35.96% of the sampled *Rattus norvegicus* and 13.33% of *R. tanezumi*. Based on the comprehensive analyses of large (L), medium (M), and small (S) segments, our study first demonstrated that the genetic characterization of urban SEOV was shaped by high nucleotide substitution rates, purifying selection, and recombination. Additionally, we detected mutational saturation in the S segment of SEOV, which may lead to the biases of genetic divergence and substitution rates in our study. Importantly, we have filled the gap of SEOV evolution in the urban area. The genetic variation of SEOV may highlight the risk of HFRS, which merits further investigation.

## 1. Introduction

Seoul virus (SEOV), which belongs to the genus *Orthohantavirus*, family *Hantaviridae*, and order *Bunyavirales*, is enveloped, single-stranded, and a negative-sense RNA virus. The virus establishes a persistent infection, which causes no apparent harm in rodents [1,2]. However, SEOV is pathogenic, which caused hemorrhagic fever with renal syndrome (HFRS) in humans [3,4]. As the major endemic countries of HFRS, China accounts for 90% of total HFRS cases worldwide [4,5,6,7]. HFRS mainly occurred in rural areas in the past, but recently extended to urban areas and even to city centers in China [4,8,9]. Unlike other hantaviruses, SEOV has a worldwide geographic distribution range, from Asia to Africa, Europe, and both Americas mainly because of worldwide spread of brown rats [10]. Under the fast socioeconomic development and frequent trade, SEOV is probably the most important global hantavirus, which posed threats to public health in most areas [10,11]. However, SEOV remains the globally most underestimated pathogenic hantavirus [11].

The SEOV genome consists of three separate segments, which is referred to as the large (L), medium (M), and small (S) segments. These three segments encode the viral RNA-dependent RNA polymerase (RdRP), two envelope glycoproteins (Gn and Gc), and nucleocapsid protein (N), respectively [12,13]. Generally, RNA viruses evolve with remarkable rapidity, high rates of mutation and substitution, short generation time, and huge population sizes [14,15]. Further evidences also show that purifying selection is a dominant evolutionary force for some RNA viral genes [14,15]. Additionally, recombination and reassortment might also play an important role in RNA virus evolution [16,17,18]. Despite of some reports on hantaviruses prevalence and evolution [19,20,21,22], the sequence information and mechanisms of evolution in SEOV, especially in urban context, remain to be fully elucidated.

Urbanization is a global trend that results from economic development [9,23]. Features of urban environments provide unique opportunities for the rats to colonize, for example, suitable harborage, food and water sources, and efficient traffic systems [24]. Additionally, close contacts occur between commensal rats and people in the urban environment, which further increases the risk of disease transmission [9,24,25,26]. Guangzhou has a population of 14.5 millionpeople, of which 20%–30% are floating (such as the temporary resident population, registered passenger population, and transit population). Indeed, the epidemic tendency of HFRS recently increased in Guangzhou [27,28]. For the first time we attempted to analyze the longer sequences of SEOV in the urban environment. By using different methods, we can gain more insights into the evolution of SEOV, which will provide effective guidance for relevant government departments.

## 2. Materials and Methods

### 2.1. Ethics Statement

The sample collection and handling complied with the Institutional Guidelines for Animal Use and Care at the Institute of Zoology, the Chinese Academy of Sciences, China (approval number IOZ12017).

### 2.2. Samples

**Figure 1:**
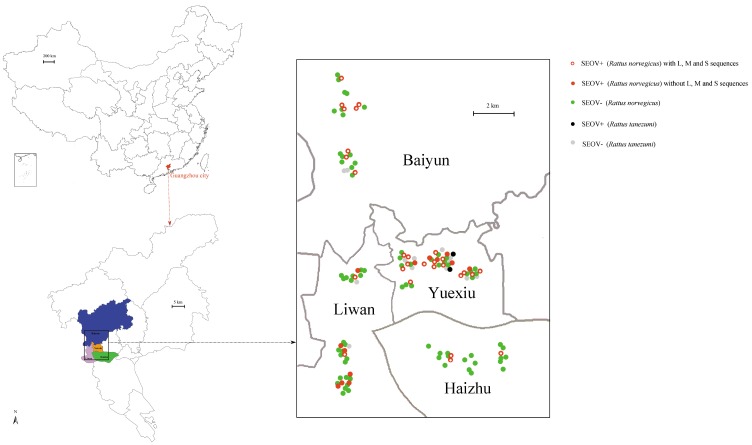
Sample collection sites, positive and negative rat distributions in Guangzhou city. Sample collection sites were represented by dots colored according to the infection status. Map was obtained from the Chinese Academy of Surveying & Mapping.

The study area comprised four districts (Haizhu, Liwan, Yuexiu, Baiyun) in Guangzhou, the capital city of the Guangdong province. Rodents are captured using both animal live traps and snap traps in the urban residential area from 2018 to 2019 (Figure 1). The four districts are old urban centers, and the population density is relatively large. The rats trapped by live traps were dissected under anesthesia and then killed for tissues sampling. The rats captured by snap traps were just processed for tissues collections. Sex and body weights were recorded. The animal species was determined by morphological identification and cytochrome b (Cyt-b) gene sequencing. The lung and liver were frozen with liquid nitrogen and then stored at −80 °C for further hantavirus detection and species identification, respectively.

### 2.3. Sequences Amplification and Sequencing

Total RNA was extracted from the lung with TRIzol (Invitrogen, Carlsbad, USA) according to the manufacturer’s instruction. A genomic DNA extraction kit (TIANGEN, Beijing, China) was used to extract DNA from the liver according to the manufacturer’s protocol. Both the quantity and quality of total RNA and DNA were assessed by measuring OD260/280 ratios using a NanoDrop ND-1000 spectrophotometer (NanoDrop Technologies, Wilmington, USA). The cDNA was synthesized in a 40 µL reaction volume by using the GoScript Reverse Transcription System (Promega, Madison, USA). A nested RT-PCR assay was used to detect currently known and possible novel members of hantaviruses associated with rodents or insectivores from the lung [29]. The sequence of the rat Cyt-b gene was recovered from the liver using a standard PCR with primers CB1 and CB2 [30]. The obtained positive fragments of hantavirus (423bp) and Cyt-b gene sequences were all successfully sequenced using the ABI 3730XL DNA Analyzer (Appled Biosystems, Foster City, USA).

The L, M, and S segments of all the PCR positive lung tissues were generated by RT-PCR. Primers were designed, according to Seoul *orthohantavirus* sequences published in GenBank, to amplify overlapping regions of the L, M, and S segments (Table S1). The PCR program was as follows: a cycle of 95 °C for 5 min, 35 cycles of 95 °C for 30 s, Tm for 30 s, 72 °C for 1 min, and then 72 °C for 7 min. The obtained amplicons were gel-purified and sequenced using the ABI 3730XL DNA Analyzer. The sequencing was repeated two–three times to ensure accuracy. Sequence editing and assembly were performed using DNAMAN8 (LynnonBioSoft, USA). The results were then submitted to the NCBI database.

### 2.4. Phylogenetic Analyses

To investigate the evolutionary relationships between SEOV from Guangzhou and other areas, we built phylogenetic trees using the L (accession numbers, MN022844-MN022871), M (accession numbers, MN022816-MN022843), and S (accession numbers, MN022788-MN022815) sequences (Table S2). Other representative sequences of SEOV were downloaded from GenBank (Table S3). The best fit substitution model was determined by jModelTest version 2.1.7 [31]. Maximum clade credibility (MCC) trees were constructed by the BEAST v1.8.4 software [32]. Different combinations of relaxed-clock models (exponential and lognormal models) and branch rate models (constant, Bayesian Skyline, extended Bayesian Skyline, and BayersianSkyGrid models) were performed to determine which was the best phylodynamic models. Then we imported the resultant *log files into Tracer (v1.6) and performed model comparison analyses (AICM analysis), and the lower AICM value was selected as the better model fit. MCC trees were obtained from the Markov Chain Monte Carlo (MCMC) tree samples using TreeAnnotator v1.8.4 with a posterior probability greater than 0.7 [32]. The resulting trees were edited and illustrated using FigTree v1.4.2 [33].

We first searched and checked 25 SEOV strains with three genome sequences in Genbank. However, three of them were not suitable for our analysis. Two of them were vaccine strains. The only obtainable host information for another one was cell. Ultimately, we chose the remaining 22 strains for L, M, and S segments genetic analyses. Then, the first 210 gene sequences in the basic local alignment search tool (BLAST) output were collected for M and S segments. The subsequent outputs after the first 210 sequences presented either low identity or low score (probably not SEOV). Notably, the number of SEOV with full L segments was limited (only 26 sequences could be used), so the number of L segments used for phylogenetic tree and evolution analysis were less than M and S segments. The initial trees of M and S segments were constructed using MEGA5 [34], and some sequences were deleted without impairing the topological structure of phylogenetic trees. The major lineages and the most basal sequences in clades were preserved [35].

In this study, a Bayesian tree (Figure S1) was constructed based on the part of the L segment amplified by the identification primers [29] with MrBayes 3.2.6 software [36]. The resulting trees were visualized in FigTree v1.4.2 [33].

### 2.5. Evolutionary Rates and Divergence Times

To evaluate the rates of nucleotide substitutions and the estimated divergence time of each gene segment from its time to the most recent common ancestors (TMRCA), we used an uncorrelated relax-clock Bayesian Markov chain Monte Carlo method in BEAST v1.8.4, with uncertainty in all estimates reflected in the 95% high probability density (HPD) intervals [32]. For each analysis, we set MCMC chains to run 100 million or 200 million iterations and sampled every 10,000 or 20,000 iterations after a 10% burn-in, and all the effective sample sizes were well over 200 [32].

Molecular data sampled over a short timeframe often appear to evolve at higher rates than those sampled over a longer time period [37,38]. Additionally, a report showed that mutational saturation was the predominant cause of biases in molecular clock dating for highly diverged hantavirus sequences [39]. So we detected the ratio of transition to transversion mutations (ti/tv), the ratio of the number of nonsynonymous to synonymous substitutions per site (dN/dS), the α shape parameter of the Γ-distribution of among-site rate variation, and GC-content through CODEML in PAML v4 [40,41]. We investigated signals of mutational saturation among different evolutionary levels, by selectively removing the more divergent lineages as the reduced evolutionary age [38,39]. Then we compared the complete and reduced data set to determine any changes in ti/tv over time. For the reduced data set, basal branches of the topology were illustrated in Supplementary Figures S2–S4.

In addition, pairwise genetic divergence was calculated in MEGA 6 [34]. Statistical significance of the correlation between the matrices of geographical distance and genetic divergence was determined with the Mantel test using the ade4 package [42].

### 2.6. Molecular Diversity and Natural Selection Analyses

Basic measures of sequence diversity, the number of variable sites (SNPs), the number of haplotype, haplotype diversity, nucleotide diversity per site (π), and Watterson’s estimator θ_W_, were summarized in DnaSP v.5 [43]. Several neutrality tests, such as Tajima’s D [44], Fu-Li D* and Fu-Li F* [45], and the McDonald-Kreitman (MK) test [46] were performed on sequence data to examine evidences for selective pressure in DnaSP v.5 [43]. The ω (dN/dS) of SEOV strains in Guangzhou was detected with PAML v4 [40,41]. For a conservative detection of sites evolving under positive selection within SEOV from Guangzhou, three segments were tested under the M7/M8 site model by CODEML in PAML v4. Models were evaluated under a likelihood ratio test (LRT) using χ^2^ distribution. Sites under positive selection were identified by Bayes’ Empirical Bayes analysis implemented in PAML v4 [40,41]. Next, a series of maximum likelihood methods were implemented in the Datamonkey Web server [47] to detect positive selected codons, such as FUBAR, FEL, and SLAC. Only sites identified by at least two of these methods were considered positively selected.

### 2.7. Detection of Recombination

Analyses were conducted on the three segments separately and also on concatenated L, M, and S segments for each isolate to detect recombination and reassortment, respectively, in the Recombination Detection Program 4 (RDP4) [48]. The presence of reassortment events was determined by applying recombination analysis techniques and then identified breakpoints located at the end of a segment. Thus, the location of breakpoints was used to distinguish recombination or reassortment events. A confirmed recombination/reassortment event was required to satisfy the following two conditions (as suggested by the RDP manual): (1) confirmed by at least two methods supported with *P* < 0.05 and (2) the RDP recombination consensus score (RDPRCS) was >0.60. If an event met the first condition, but had an RDPRCS in the range 0.4–0.6, it was considered a possible recombination; otherwise the event was rejected. The plausible recombination/reassortment events were shown in the table, which listed detection methods and significance, RDPRCS values, and breakpoints. The estimation of recombination breakpoint confidence intervals was used to build new phylogenetic trees by Bayesian methods using MrBayes 3.2.6 software [36]. The resulting trees were visualized in FigTree v1.4.2 [33].

### 2.8. Statistical Analysis

The differences of prevalence between species, sex, and body weights were analyzed through Mann-Whitney U Test in SPSS16.0 (IBM, New York, USA). A probability of 0.05 was considered to be the threshold.

## 3. Results

### 3.1. Samples and Prevalence Rate

A total of 129 animals were obtained in the four districts in Guangzhou city. Males (*n* = 67) were trapped slightly more than females (*n* = 62). *R. norvegicus* (*n* = 114) were trapped more than *R. tanezumi* (*n* = 15). Cyt-b sequences obtained from liver confirmed the species. Lung samples were tested for the presence of hantavirus RNA with primers directed at a conserved region of L segment. Sequencing and BLAST in NCBI confirmed the presence of SEOV RNA in 43 of 129 samples, which lead to a positive rate of 33.33% (Figure 1). The positivity rate was 35.96% (41/114) and 13.33% (2/15) in *R. norvegicus* and *R. tanezumi*, respectively (Figure 1). The positive rates showed no significant differences in species and sexes (Table 1). In addition, the weights of positive rats were significantly heavier than those of negative ones (Table 1).

### 3.2. Phylogenetic Analyses

A total of 28 partial L segment sequences (nt36–6323), 28 full CDS sequences of M segment (nt 46–3447), and 28 partial S segment sequences (nt 429–1331) were successfully obtained from PCR-positive *R. norvegicus*. We did not obtain the virus sequences from tissues of positive *R. tanezumi* (Figure 1). Based on the Bayesian tree of the part L segment amplified by the identification primers, no significant divergence was found between these successful or failed amplified SEOV (Figure S1).

**Figure 2:**
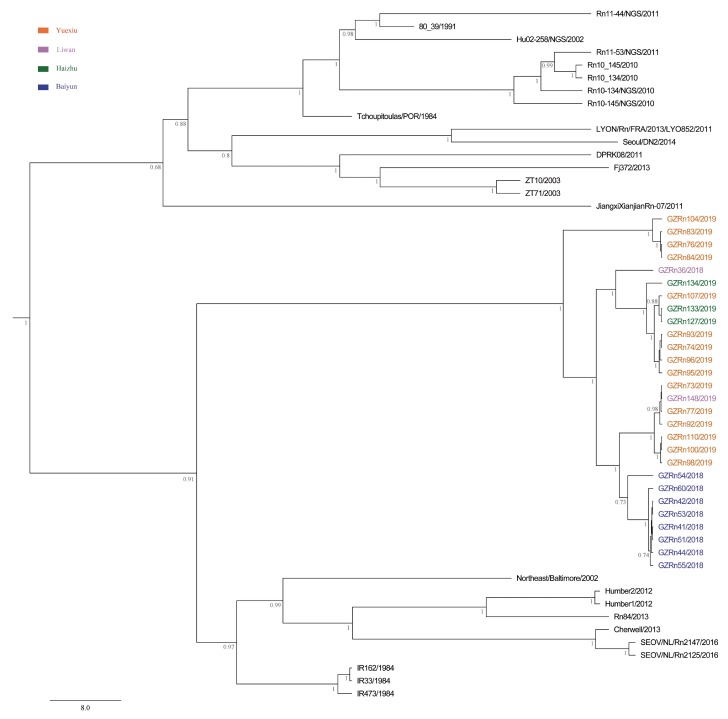
Maximum clade credibility (MCC) tree of the large (L) segment in SEOV in Guangzhou. The tree was built using BEAST v1.8.4 and illustrated using FigTree v1.4.2. Posterior probabilities of major nodes were indicated.

**Figure 3:**
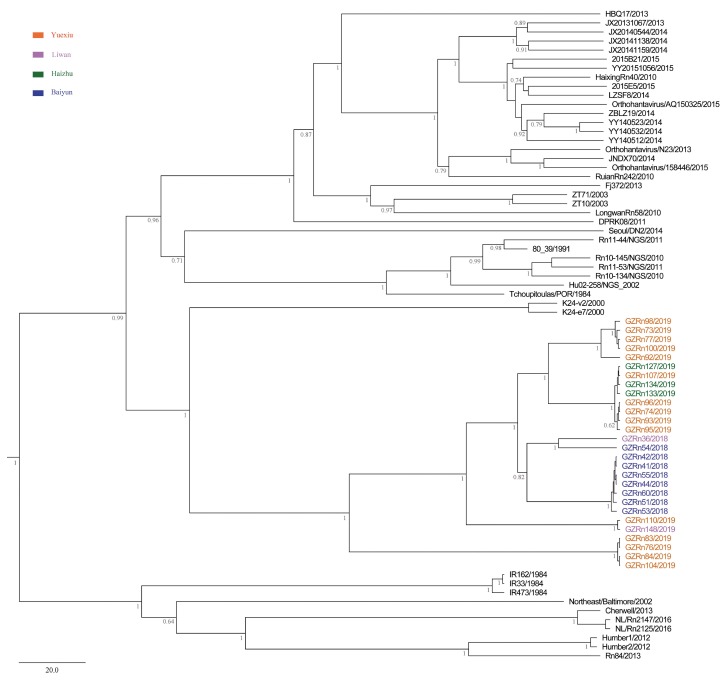
MCC tree of the medium (M) segment in SEOV in Guangzhou. The tree was built using BEAST v1.8.4 and illustrated using FigTree v1.4.2. Posterior probabilities of major nodes were indicated.

**Figure 4:**
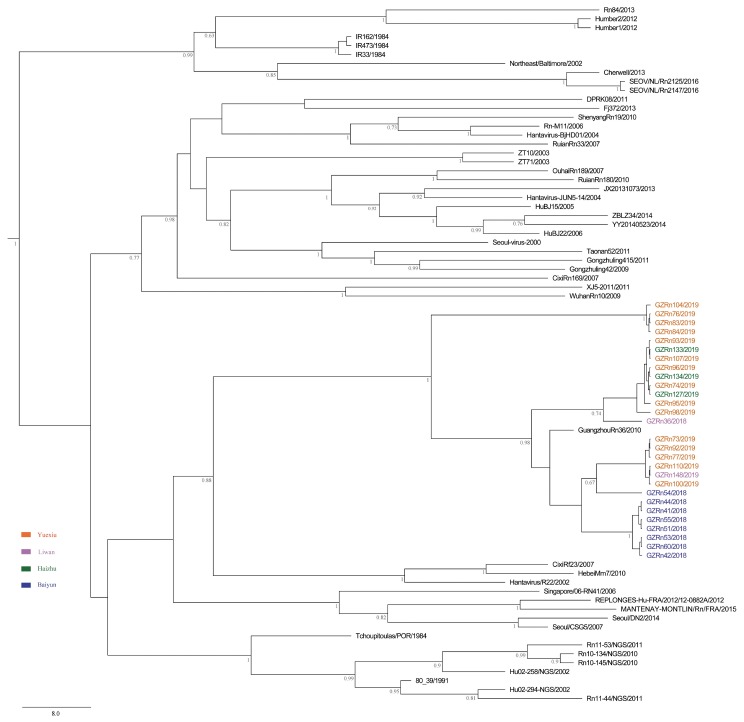
MCC tree of the small (S) segment in SEOV in Guangzhou. The tree was built using BEAST v1.8.4 and illustrated using FigTree v1.4.2. Posterior probabilities of major nodes were indicated.

The MCC trees showed that Guangzhou SEOV clustered together in a single well-supported clade, indicating the genetic specificity of SEOV in Guangzhou (Figure 2, Figure 3 and Figure 4). Moreover, the strains from Baiyun District clustered together for L segment. In addition, we did not find any significant association between geographical distance and genetic divergence in Guangzhou SEOV (all *P* > 0.5).

### 3.3. Evolutionary Rates and Divergence Time

Former reports showed that failure to account for mutational saturation can lead to an increase in the estimate of α over time and a decline in that of ti/tv [37,38]. Our results showed that estimates of α increased while estimates of ti/tv declined over time, indicating that the substitution model did not accurately account for mutational saturation in the complete data sets for S segment. This may cause the biases in estimates of the substitution rate and divergence time in the S segment (Table 2 and Table 3). The ti/tv of L and M segments did not show decline over time (Table 4). Additionally, no evidences of temporal differences in GC content were observed in L, M, and S segments (Table 4). Nucleotide length, best-fit model, and MCMC chain length for each segment of SEOV were displayed in Table S4.

### 3.4. Molecular Diversity and Natural Selection Analyses

The SNPs identified from L, M, and S segments of SEOV in Guangzhou were 214, 160, and 33, respectively. The numbers of haplotype monitored in the L, M, and S segments were 18, 20, and 7, respectively. Haplotype diversity values across loci ranged from 0.815 to 0.960, and nucleotide diversity values ranged from 0.010 to 0.014 (Table 5). The values of Tajima’s D, Fu-Li D*, and Fu-Li F* were negative, but the levels did not significantly deviate from neutral expectations (Table 5). We used Seoul/DN2/2014 that was divergent from Guangzhou SEOV strains as an outgroup for MK test in all the three segments. We detected strong purifying selection in the L segment of Guangzhou SEOV, as the neutrality index value above 1 indicated an excess of replacement changes (Table 5). The selection pressure analyses of Guangzhou SEOV revealed that ω values ranged from 0.035 to 0.049 (far lower than 1), indicating that L, M, and S segments tended to be subjective to purifying selection (Table 6). Additionally, codon-specific analyses also indicated some evidences of positive selection (Table 6). One positively selected site (codon 66) in the L segment was significant with PAML and FUBAR (Table 6).

### 3.5. Recombination Analysis

In recombination analysis, six significant potential recombination events (PRE) were detected in L (GZRn95, 98, and 104) and M (GZRn92, 98, and 110) segments (Table 7). The breakpoints of these PREs were listed in Table 7. We constructed the phylogenetic trees using sequences of either the putative recombinant region or the region without recombination. Significant changes in the topology of the trees were observed in GZRn98 and GZRn104 of the L segment, and in GZRn92 and GZRn110 of the M segment. Notably, GZRn95 and GZRn104 with different haplotypes had the same recombination area and they were both obtained from Yuexiu District. In addition, GZRn98 was actually a double recombinant, because it had recombination events in both L and M segments. (Figures S5–S10). However, no reassortment was detected in our analysis.

## 4. Discussion

Numerous HFRS outbreaks are major environmental and public health concerns in the world, especially in developing countries [9,11,23]. Although efforts have been made, the paucity of SEOV sequence data has hampered important researches. Here we implement an in-depth analysis of the genetic diversity and molecular evolution of SEOV in the urban environments.

The epidemiology investigation revealed that SEOV was the main hantavirusin Guangzhou city. This result was in agreement with former reports, which indicated that SEOV was predominant in the residential habitats in China [4,10,11]. The amount of captured *R. norvegicus* was higher than *R. tanezumi*, suggesting that *R. norvegicus* was the dominant species in Guangzhou city. However, the prevalence did not significantly differ between these two species. Our results confirmed that *R. norvegicus* was the predominant carriers for hantaviruses in the residential habitats [10,11]. The high prevalence (33.33%) of SEOV may be directly related to the reservoir species and urban environment [10]. Due to a combination of adequate harborage, food, and water supply, the population of city rats was high [23,24,25]. Moreover, the frequent contact and dispersal of city rats might increase the spread of the virus, as we did not find any significant associations between geographical distance and genetic divergence in Guangzhou SEOV. Generally, weight is a good proxy for age [49,50]. The weights of positive ratswere significantly heavier than those of negative ones, which demonstrated that the older the animal, the greater the likelihood that it will become exposed to and infected with SEOV [51]. Moreover, body weight has been used as an indicator of sexual maturity in *R. norvegicus*. It meant that physiological and behavioral changes associated with sexual maturation may favor SEOV infection [51].Unfortunately, we failed to recover the L, M, and S segments from *R. tanezumi* and the other PCR-positive *R. norvegicus* probably due to the sample quality. The rats in our study were trapped through snap traps and live traps. Thus, both dead and live rats were captured. We put snap traps the night before, checked and collected animals early in the next morning. Thus, the rats might be caught early and were dead for a long time, which caused the virus to degrade, further interfered with the amplification of gene segments. SEOV was a RNA virus, and thus the amplification of genome segments required high quality of tissues. Report shows that hantavirus is sensitive to heat (30 min at 60 °C) [52] and the longevity of Puumala virus (PUUV) and Tula virus (TULV) depends on the temperature and moisture [53]. In nature, there are additional physical and chemical factors, such as UV light, sunlight, and pH, which may influence the longevity of viruses [53]. Moreover, no significant divergence was found between these successful or failed amplified SEOV on the basis of the part L segment. Particularly, we also recovered part of L, M, and S segments in other positive rats. The sequences were short and thus we did not use them for evolutionary analysis. *R. norvegicus* and *R. tanezumi* are partially sympatric in southern China [54]. However, over the past 20 years, *R. tanezumi* has significantly expanded northward in China and partially replaced the native *R. norvegicu* [54]. It will be a good example to clarify the adaptive evolution strategy of SEOV in southern and northern China, thus advancing the research of this severe human pathogen.

In our study, the best-fitting time-reversible model of nucleotide substitution was used for each gene segment to minimize the impact of site saturation. However, we still detected mutational saturation in the S segment of SEOV. Former studies showed that high levels of saturation were attained extremely rapidly in RNA virus [35,36]. Mutational saturation in the S segment may lead to incorrect estimation of evolutionary rates and time-scales in our study. For example, Saxenhofer et al. [37] observed strong signals of mutational saturation at different levels of genetic divergence and they demonstrated that mutational saturation was the predominant cause of biases in molecular clock dating in PUUV and TULV [37]. The mean rate of evolutionary change in SEOV was 10^−4^ substitutions/site/year, which was similar to the rate of other RNA viruses [15,19,21,55]. For instance, the median substitution rate of PUUV was estimated as 2.70 × 10^−4^ substitutions/site/year [21]. Such a high substitution rate may be associated with the fact that SEOV relies on RNA-dependent RNA polymerase for replication, and lacks proofreading or base-excision repair mechanism [14,15]. Generally, the substitution rate is best described as the long-term rate at which genetic variants become fixed over evolutionary timescales. Hence, this rate reflects the complex interplay of natural selection, genetic drift, modes of transmission, and epidemiological processes [14,15], although sometimes displays a time-dependent bias in rate estimates [35,36,37]. Conclusively, these observations suggest that urban SEOV is evolving substantially as fast as most RNA viruses.

A better understanding of mechanisms that drive viral genetic variation may help us to understand the epidemiology [14,15,19]. The molecular evolution of SEOV has long been overlooked. We found a high level of nucleotide polymorphisms in Guangzhou SEOV. The ratios of nonsynonymous to synonymous substitutions were very low for L, M, and S segments, indicating that most amino acid changes were deleterious, which would be removed by purifying selection. Thus, amino acid residues are preserved, while allowing near-neutral nucleotide sequences to continue evolving. Previous studies also revealed negative selection in other viruses [56,57,58]. The positive selected codon 66 in the L segment may play an important role among these SEOV recovered from Guangzhou. For TULV in a natural hybrid zone, Saxenhofer et al. (2019) pointed to the positive selected codon 17 in the M segment [59]. These positive selected sites may provide potential basis for the virus evolution. Phylogenetic analyses showed that Guangzhou SEOV clustered together in a single well-supported clade, indicating the genetic specificity of SEOV in Guangzhou. Environmental heterogeneity may shape the evolution of SEOV in Guangzhou. For example, phylogenetic analyses of PUUV typically detect genetic divergence even at relatively small geographical distances [21]. This maybe because RNA viruses exert high evolutionary rates and genetic variability, and those variants which are adapted to different localities will be selected and fixed [21,60].

For many RNA viruses, recombination has been an important feature of their evolution [16,17]. In the present study, the genetic recombination was observed in L and M segments, and the former was first detected in nature. Moreover, GZRn95 and GZRn104 in L segment from the same sampling area had the same breakpoint, although their haplotypes were different. Particularly, GZRn98 was actually a double recombinant, because recombination was detected in L and M segments. These interesting findings are worth studying in the future. Mostowy et al. [61] reported that the “recent recombinations” are determined as those that are present in some subset of strains in a lineage, while the “ancestral recombinations” are determined as those that are present between lineages that affect all strains in the lineage [61]. We detected recombinations only in a few isolates of the Guangzhou strain. Thus, we inferred that these supposed events were recent. In addition, we detected no recombination in the S segment, which differed from another study conducted in Beijing [5]. This might be attributed to the short sequence length of S segments in our study. Classically, viruses that generate persistent rather than acute infections may have high rate of recombination [17]. Each hantavirus species associates closely with rodent species, where the virus establishes a persistent but asymptomatic infection [1,13,62]. Therefore, recombination appears to be a common occurrence in hantaviruses [17]. Recombination could lead to evolutionary changes [16,17]. For example, Xiao et al. [63] demonstrated that recombination accelerated poliovirus adaptation and evolution, and was essential to enrich the population in beneficial mutations and to purge it from deleterious mutations. The recombination of L and M segments might be beneficial for SEOV fitness in Guangzhou, yet, remains to be studied.

## 5. Conclusions

Urban environments have proven to be favorable for rat population growth and associated zoonoses transmission [9,11]. Our results indicate that the genetic diversity of SEOV is shaped by the combined effects of high evolutionary rate, purifying selection, and recombinationin Guangzhou urban area. Although the number of recovered sequences in our study is limited, our results still provide valuable information of this virus. Such high genetic diversity may have important implications, such as the emergence of SEOV with different transmission abilities or virulence [13,14]. In view of fast urbanization, our study may provide directions for future infectious disease management.

## Figures and Tables

**Table 1:** Detection of Seoul virus (SEOV) in rats from Guangzhou.

Category	Subcategory	Positive (%) *n* = 43	Negative (%) *n* = 86	*P*
Sex	Male	24 (55.81)	44 (51.16)	0.804
	Female	19 (44.19)	42 (48.84)	
Species	*R. norvegicus*	41 (95.35)	73 (84.88)	0.082
	*R. tanezumi*	2 (4.65)	13 (15.12)	
Weight (g)	Median	339.43	258.12	**

** *P* < 0.01.

**Table 2:** Estimated evolutionary rates of each gene segment of SEOV by Bayesian analysis.

Categories	Substitution Rate and 95% HPD (Substitution/Site/Year)		
	Mean	Lower	Upper
L complete data set	6.2741 × 10^−4^	3.8152 × 10^−4^	8.7360 × 10^−4^
subset I	1.5114 × 10^−3^	1.1170 × 10^−3^	1.9035 × 10^−3^
subset II	1.6653 × 10^−3^	1.1053 × 10^−3^	2.2836 × 10^−3^
M complete data set	2.0631 × 10^−4^	1.2591 × 10^−4^	2.9082 × 10^−4^
subset I	2.3236 × 10^−4^	9.9816 × 10^−5^	4.0076 × 10^−4^
subset II	2.4408 × 10^−4^	9.2609 × 10^−5^	4.4428 × 10^−4^
S complete data set	4.2126 × 10^−4^	2.1798 × 10^−4^	6.1856 × 10^−4^
subset I	3.7663 × 10^−4^	1.8639 × 10^−4^	5.9634 × 10^−4^
subset II	2.7569 × 10^−4^	1.1684 × 10^−4^	4.3243 × 10^−4^

**Table 3:** The estimated divergence time of each gene segment from its time to the most recent common ancestors (TMRCA).

Categories	Estimated Divergence Time	95% HPD	
		Lower	Upper
L complete data set	2007	1990	2015
subset I	2014	2011	2016
subset II	2013	2009	2017
M complete data set	1937	1895	1970
subset I	1942	1890	1988
subset II	1943	1877	1991
S complete data set	1993	1974	2008
subset I	1991	1969	2008
subset II	1984	1948	2004

**Table 4:** Mutational saturation parameters determined for L, M, and S segment.

Categories	N	ti/tv	dN/dS	GC	α
L complete data set	54	7.57838	0.02768	0.37401	0.13075
subset I	45	7.30520	0.02673	0.37397	0.11973
subset II	38	7.33046	0.03015	0.37359	0.05750
M complete data set	72	8.07440	0.03718	0.40137	0.15019
subset I	58	8.22977	0.03735	0.40129	0.11013
subset II	48	8.24877	0.03633	0.40147	0.10923
S complete data set	78	3.80060	0.09889	0.45790	0.52316
subset I	66	5.85686	0.05748	0.45737	0.15095
subset II	55	10.35946	0.03009	0.45689	0.03428

N—number of sequences; ti/tv—transition/transversion ratio; dN/dS—ratio of non-synonymous to synonymous substitutions per site; GC—GC-content; α—shape parameter of the gamma distribution of among-site rate variation.

**Table 5:** Genetic analyses at L, M, and S segments of SEOV in Guangzhou.

Gene	L	M	S
Number	28	28	28
Fragment size (bp)	6288	3402	903
SNP	214	160	33
Number of haplotype	18	20	7
Haplotype diversity	0.955	0.960	0.815
π	0.010	0.014	0.010
θw	0.008	0.012	0.009
Tajima’s D	−0.109	−0.099	−0.105
Fu-Li D *	−0.111	−0.092	−0.082
Fu-Li F *	−0.129	−0.116	−0.106
McDonald-Kreitman (NI)	2.212 *	2.590	1.100

* *P* < 0.05.

**Table 6:** Tests of positive selection by different methods.

Gene	Number of Segments	Test of Selection					Sites Under Selection Identified by Different Methods			
		ω	InL M7	lnL M8	2lnDL	Significance	PAML M8	FEL	SLAC	FUBAR
L	28	0.039	−10011.688	−10009.035	5.307	*	66	none	none	66, 150, 753
M	28	0.049	−5715.383	−5713.898	2.969	NS	none	none	none	90
S	28	0.035	−1426.349	−1426.350	0.003	NS	none	none	none	none

NS—not significant; none—no positive site was detected; * *P* < 0.05.

**Table 7:** Recombination statistics of L and M segments of SEOV in Guangzhou.

Gene	L			M		
Number	GZRn95	GZRn98	GZRn104	GZRn92	GZRn98	GZRn110
Breakpoint	5578, 6288	89, 903	5578, 6288	2129, 2714	550, 1731	598, 1472
RDPRCS	0.570	0.687	0.609	0.708	0.561	0.661
RDP	NS	NS	NS	NS	**	NS
GENECONV	**	**	**	**	**	**
BootScan	NS	**	NS	NS	NS	**
MaxChi	**	NS	**	NS	**	**
Chimaera	**	NS	**	NS	**	**
SiScan	**	NS	**	**	**	**
3Seq	**	NS	**	**	**	**

NS—not significant; ** *P* < 0.01.

## Supplementary Materials

The following are available online at https://www.mdpi.com/1999-4915/11/12/1137/s1, **Table S1** Primers used to amplify the L, M, and S gene segments of SEOV. **Table S2:** The GenBank accession number of L, M, and S segments recovered in this study. **Table S3:** SEOV strains and their GenBank accession number for those sequences used to create L, M, and S segment phylogenetic trees. **Table S4:** Nucleotide length, best-fit model, and MCMC chain length for each gene segment of SEOV. **Figure S1:** Bayesian tree of L segment in SEOV from Guangzhou based on the identification primers. **Figure S2:** Definition of the reduced-age data for L segment. **Figure S3**. Definition of the reduced-age data for M segment. **Figure S4**. Definition of the reduced-age data for S segment. **Figure S5:** Phylogenetic trees of recombination event involved in GZRn95 in L segment. **Figure S6:** Phylogenetic trees of recombination event involved in GZRn98 in L segment. **Figure S7:** Phylogenetic trees of recombination event involved in GZRn104 in L segment. **Figure S8:** Phylogenetic trees of recombination event involved in GZRn92 in M segment. **Figure S9:** Phylogenetic trees of recombination event involved in GZRn98 in M segment. **Figure S10:** Phylogenetic trees of recombination event involved in GZRn110 in M segment.
