# Supplementary material for: Genetic Characterization and Molecular Evolution of Urban Seoul Virus in Southern China
Source: Viruses. 2019 Dec 9;11(12):1137. doi: 10.3390/v11121137 (PMC6950471; doi:10.3390/v11121137)
Supplement: Supplementary file 1 [file viruses-11-01137-s001.zip › viruses-608608-suppl final version/Supplementary figures.pdf]

Supplementary Figures

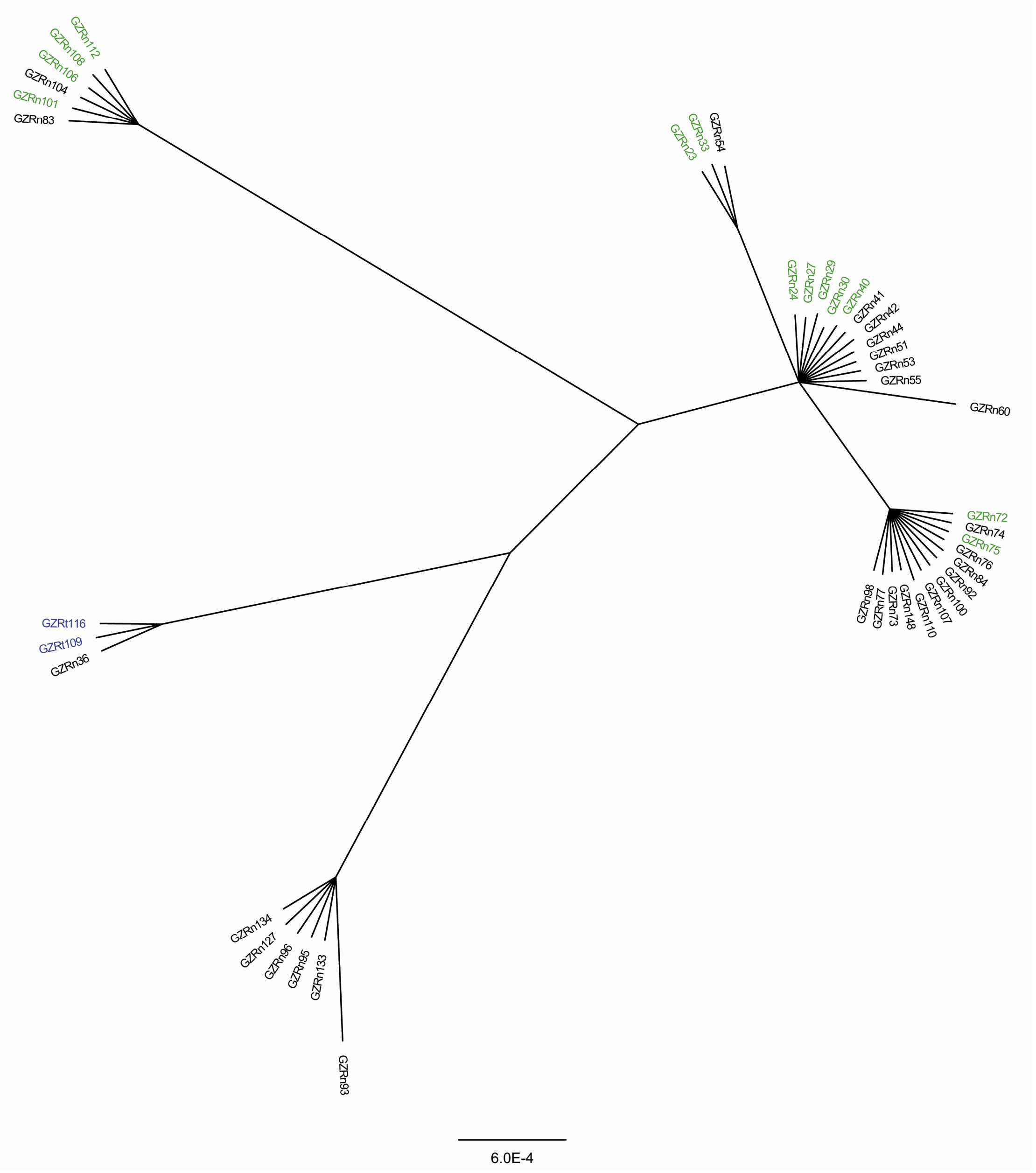

Figure S1. Bayesian tree of L segment in SEOV from Guangzhou based on the identification primers. The strains in green indicated SEOV+ (*Rattus norvegicus*) without L, M and S segment. The strains in blue indicated SEOV+ (*Rattus tanezumi*). The strains in black indicated SEOV+ (*Rattus norvegicus*) with L, M and S segment.

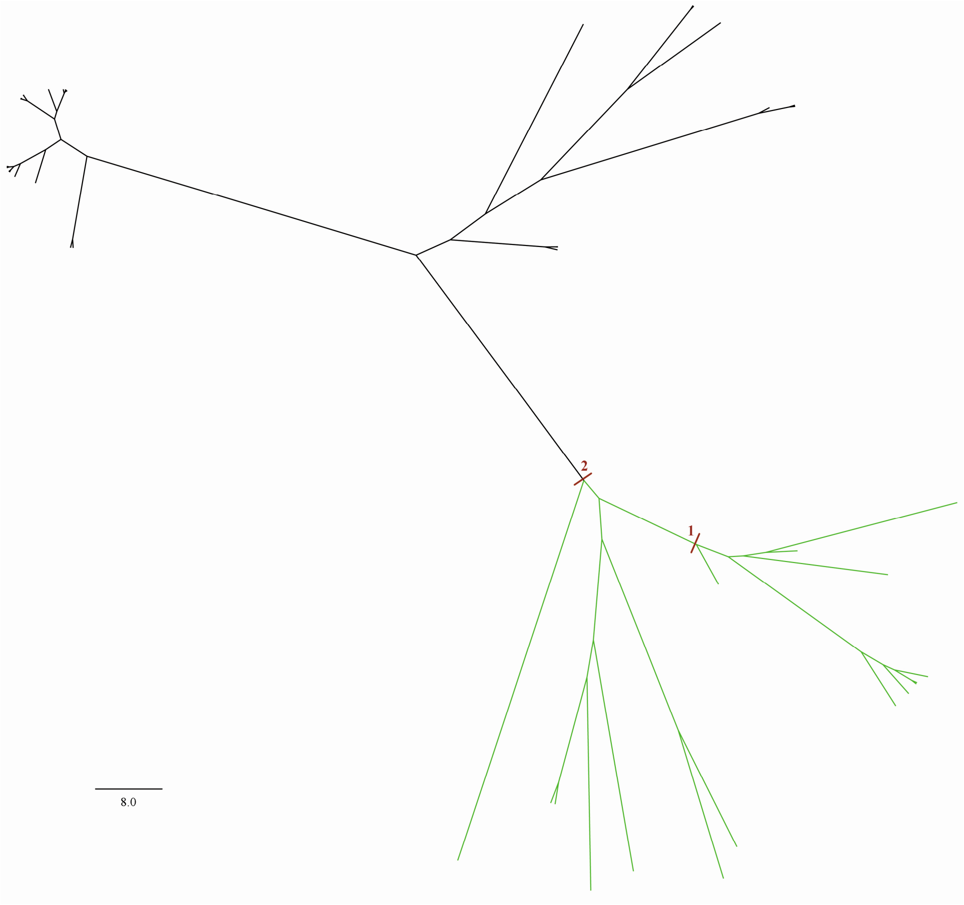

Figure S2. Definition of the reduced-age data for L segment following Duchêne et al (2015). Sequences in Mrbayes phylogeny topology connecting to basal branches (green) were removed to create a sequence subset of reduced evolutionary age (1: subset I; 2: subset II).

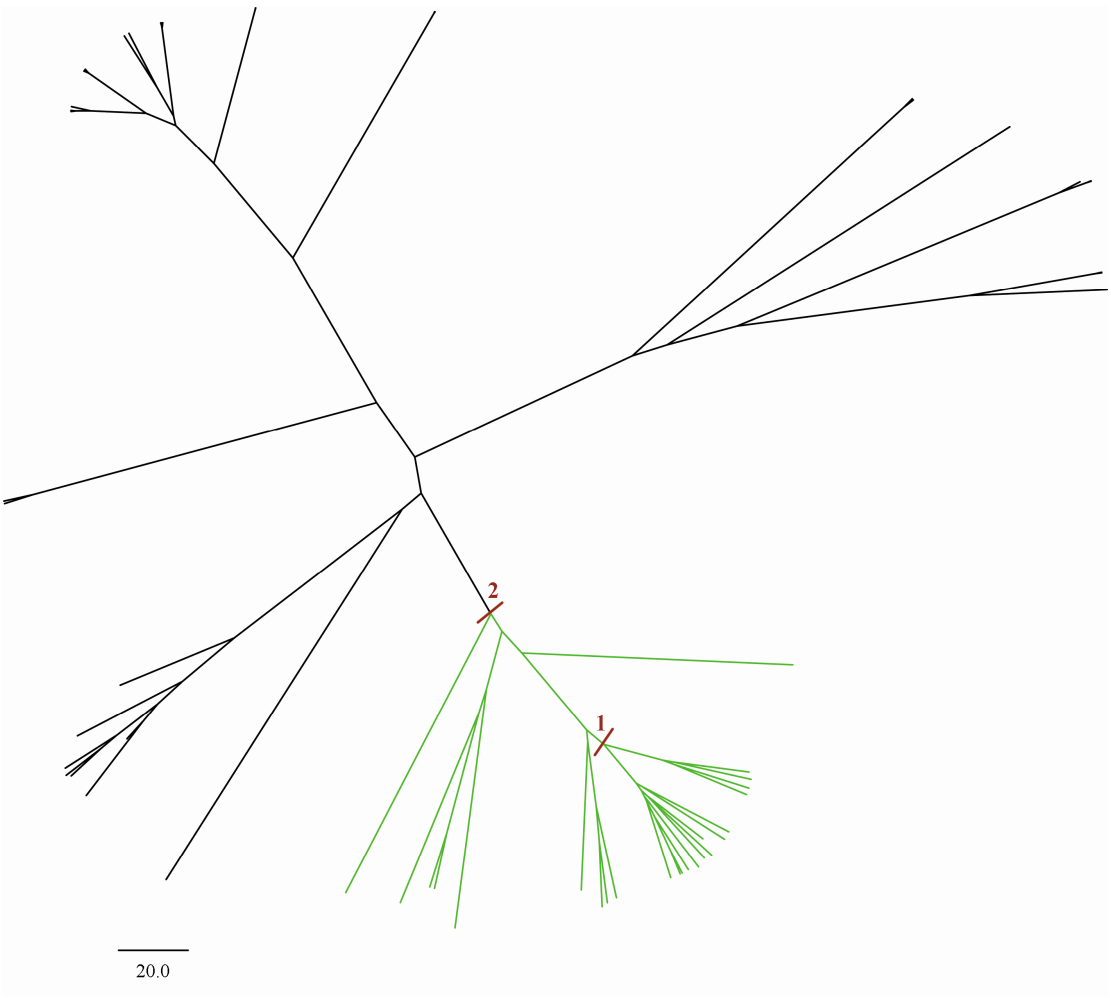

Figure S3. Definition of the reduced-age data for M segment following Duchêne et al (2015). Sequences in Mrbayes phylogeny topology connecting to basal branches (green) were removed to create a sequence subset of reduced evolutionary age (1: subset I; 2: subset II).

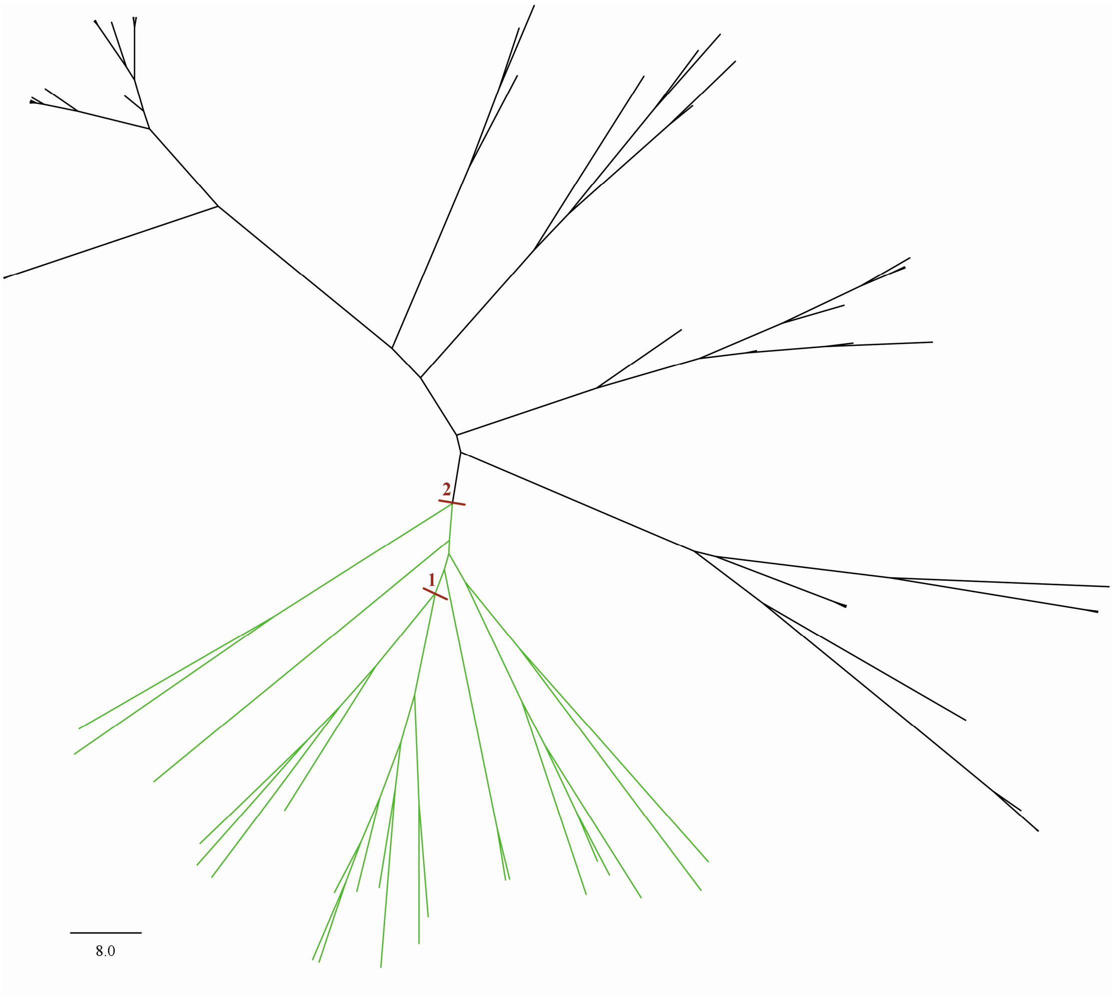

Figure S4. Definition of the reduced-age data for S segment following Duchêne et al (2015). Sequences in Mrbayes phylogeny topology connecting to basal branches (green) were removed to create a sequence subset of reduced evolutionary age (1: subset I; 2: subset II).

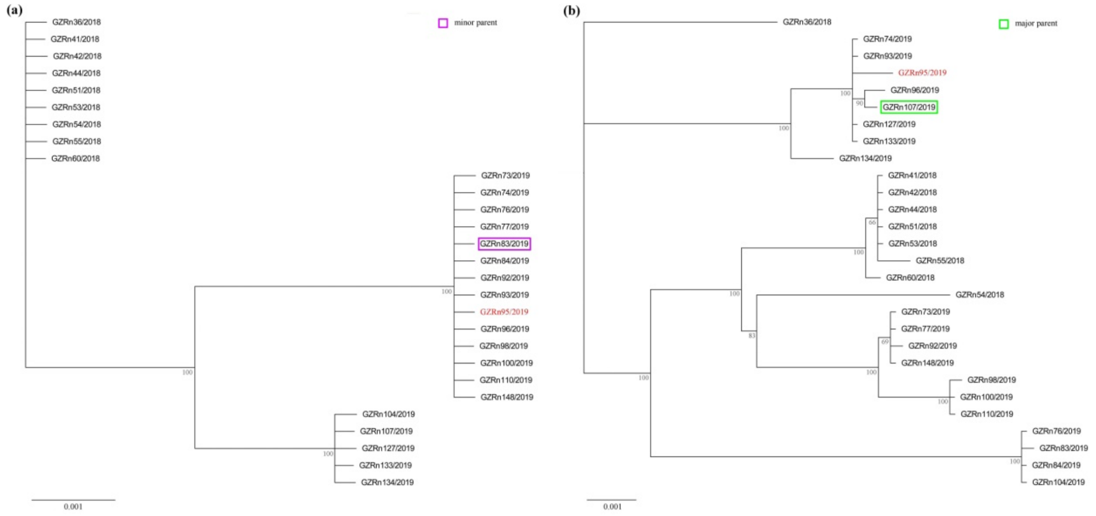

Figure S5. Phylogenetic trees of recombination event involved in GZRn95 in L segment. (a): Phylogenetic tree based on 5578-6288 nucleotide of the L segment. (b): Phylogenetic tree based on concatenated sequence of the remainder of our sequence alignment. Recombination individual was indicated in red. The major parent and minor parent were indicated in green and pink pane, respectively.

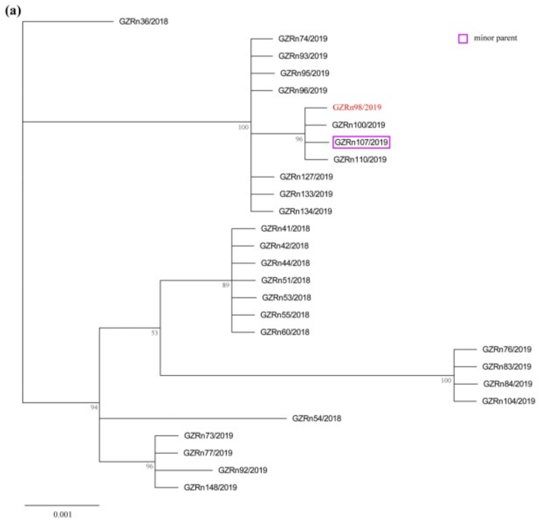

Figure S6. Phylogenetic trees of recombination event involved in GZRn98 in L segment. (a): Phylogenetic tree based on 89-903 nucleotide of the L segment. (b): Phylogenetic tree based on concatenated sequence of the remainder of our sequence alignment. Recombination individual was indicated in red. The major parent and minor parent were indicated in green and pink pane, respectively.

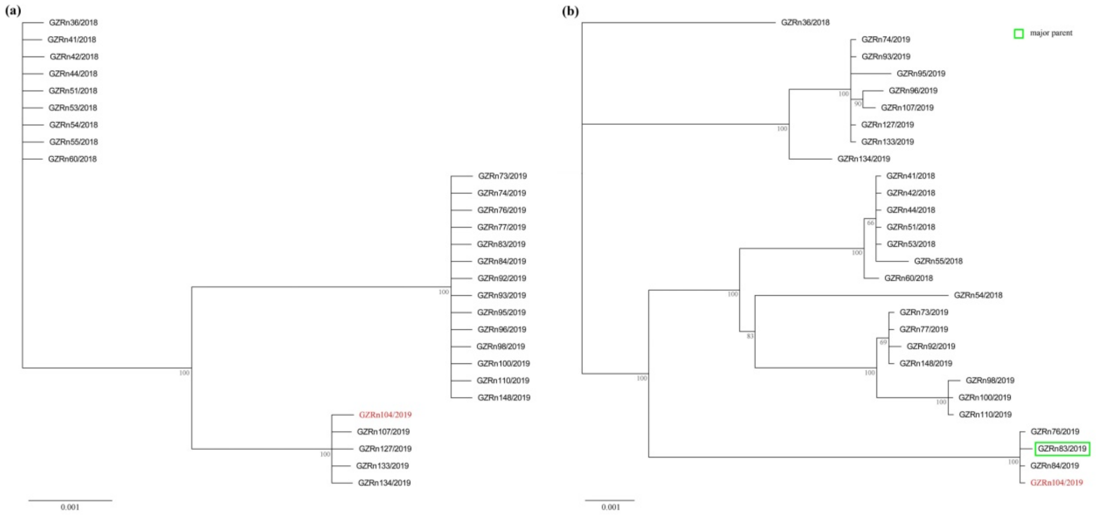

Figure S7. Phylogenetic trees of recombination event involved in GZRn104 in L segment. (a): Phylogenetic tree based on 5578-6288 nucleotide of the L segment. (b): Phylogenetic tree based on concatenated sequence of the remainder of our sequence alignment. Recombination individual was indicated in red. The major parent was indicated in green pane. The minor parent was unknown.

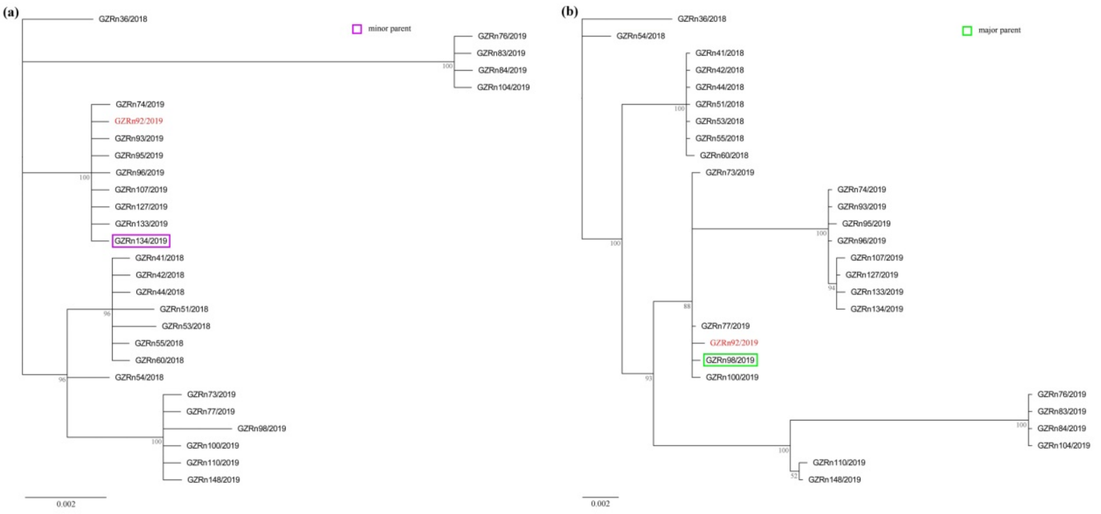

Figure S8. Phylogenetic trees of recombination event involved in GZRn92 in M segment. (a): Phylogenetic tree based on 2129-2714 nucleotide of the M segment. (b): Phylogenetic tree based on concatenated sequence of the remainder of our sequence alignment. Recombination individual was indicated in red. The major parent and minor parent were indicated in green and pink pane, respectively.

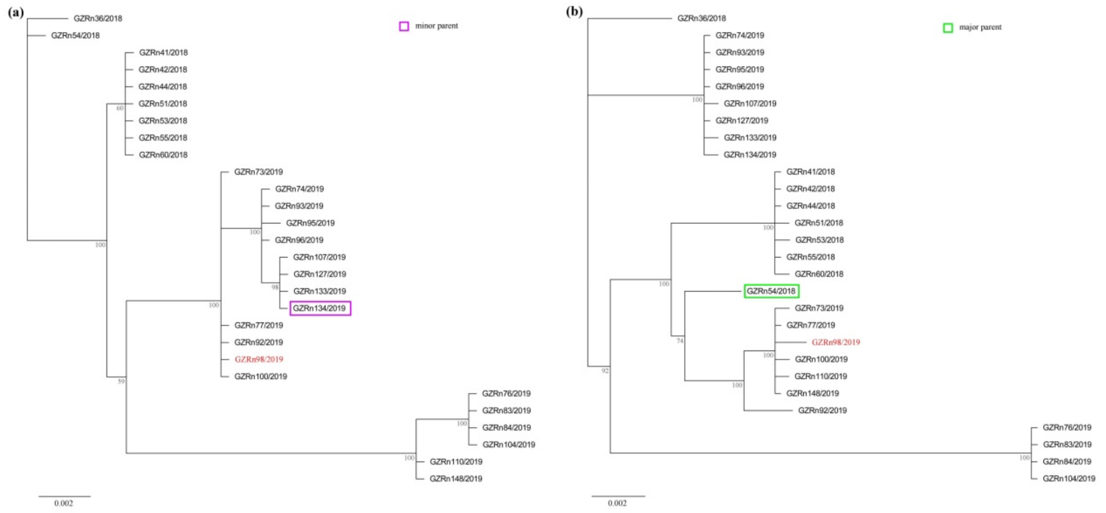

Figure S9. Phylogenetic trees of recombination event involved in GZRn98 in M segment. (a): Phylogenetic tree based on 550-1731 nucleotide of the M segment. (b): Phylogenetic tree based on concatenated sequence of the remainder of our sequence alignment. Recombination individual was indicated in red. The major parent and minor parent were indicated in green and pink pane, respectively.

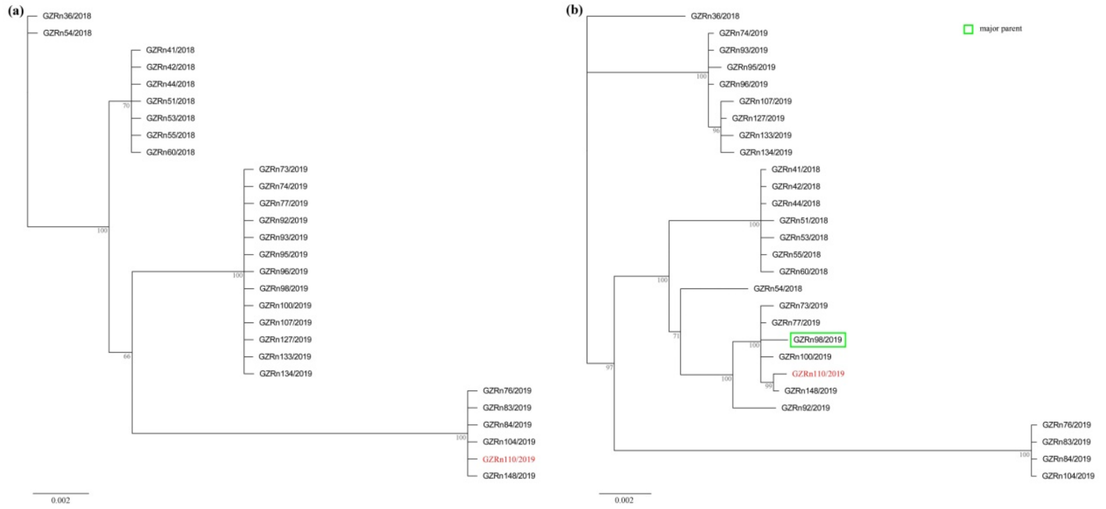

Figure S10. Phylogenetic trees of recombination event involved in GZRn110 in M segment. (a): Phylogenetic tree based on 598-1472 nucleotide of the M segment. (b): Phylogenetic tree based on concatenated sequence of the remainder of our sequence alignment. Recombination individual was indicated in red. The major parent was indicated in green pane. The minor parent was unknown.
