# Supplementary material for: Genetic Characterization and Molecular Evolution of Urban Seoul Virus in Southern China
Source: Viruses. 2019 Dec 9;11(12):1137. doi: 10.3390/v11121137 (PMC6950471; doi:10.3390/v11121137)
Supplement: Supplementary file 1 [file viruses-11-01137-s001.zip › viruses-608608-suppl final version/Supplementary tables.pdf]

Supplementary Tables

Table S1 Primers used to amplify the L, M, and S gene segments of SEOV

Primer name	Sequence 5'-3'	Tm (℃)	Products (bp)
Seoul-LF1	GGAAGAGACAAATCAAAAA	50	835
Seoul-LR1	AAAACAATCAAACACAGCT		
Seou-LF2	AACCTCATCCCAGAAACCC	58	967
Seou-LR2	CAACCAAAGAAGGAAAGCC		
Seoul-LF3	TTCTGGCTTTCCTTCTTTGG	62	768
Seoul-LR3	CCTCGATCAGCTTCTGTCCT		
Seoul-LR4	ACAAGCTAGAATTGTGAGG	54	1047
Seoul-LF5	TGTGAGATTTGAGGTGATG		
Seoul-LF5	TTTTGAGGGTTGTGCTGTA	56	1067
Seoul-LR5	GAGAATCTGGGAACTTTGC		
Seoul-LF6	GGGGAATGTGGAAGGAAC	56	846
Seoul-LR6	GCTAAAAGAAACCGTGGG		
Seoul-LF7	CGGTTTCTTTTAGCACAT	54	740
Seoul-LR7	ACAACAGACTCACCCCAT		
Seoul-MF1	GCAGGATCATGTGGAGTT	52	620
Seoul-MR1	TATGCTTGCTATGGCGTA		
Seoul-MF2	GCTGTTTGATTGGTCTTGGT	58	1134
Seou-MR2	CTCTTGATTGCTTGTGTGGA		
Seoul-MF3	TTGAAAAAACAAAGGGTTC	56	1005
Seoul-MR3	CTCCAAAGTGACAGGTAGA		
Seoul-MF4	ATGAGAGAATTGAATGGAG	52	755
Seoul-MR4	AAGATTGGGAGATAGAAGA		
Seoul-SF1	TAACATCATTCGTGGTCCC	56	471
Seoul-SR1	CTTGAAATTCCTTTGGCTC		
Seoul-SF2	AGTGTAGTTGGGTTTTTGG	54	1003
Seoul-SR2	AAGGTTGTGAGGTAGTTTG		

Table S2 The GenBank accession number of L, M, and S segments recovered in this study

Seoul virus	Area (district)	Host sex	Accession number		
			L segment	M segment	S segment
GZRn36	Liwan	Male	MN022844	MN022816	MN022788
GZRn41	Baiyun	Female	MN022845	MN022817	MN022789
GZRn42	Baiyun	Male	MN022846	MN022818	MN022790
GZRn44	Baiyun	Female	MN022847	MN022819	MN022791
GZRn51	Baiyun	Male	MN022848	MN022820	MN022792
GZRn53	Baiyun	Male	MN022849	MN022821	MN022793
GZRn54	Baiyun	Female	MN022850	MN022822	MN022794
GZRn55	Baiyun	Female	MN022851	MN022823	MN022795
GZRn60	Baiyun	Female	MN022852	MN022824	MN022796
GZRn73	Yuexiu	Male	MN022853	MN022825	MN022797
GZRn74	Yuexiu	Male	MN022854	MN022826	MN022798
GZRn76	Yuexiu	Male	MN022855	MN022827	MN022799
GZRn77	Yuexiu	Male	MN022856	MN022828	MN022800
GZRn83	Yuexiu	Female	MN022857	MN022829	MN022801
GZRn84	Yuexiu	Female	MN022858	MN022830	MN022802
GZRn92	Yuexiu	Male	MN022859	MN022831	MN022803
GZRn93	Yuexiu	Male	MN022860	MN022832	MN022804
GZRn95	Yuexiu	Male	MN022861	MN022833	MN022805
GZRn96	Yuexiu	Female	MN022862	MN022834	MN022806
GZRn98	Yuexiu	Male	MN022863	MN022835	MN022807
GZRn100	Yuexiu	Male	MN022864	MN022836	MN022808
GZRn104	Yuexiu	Male	MN022865	MN022837	MN022809
GZRn107	Yuexiu	Female	MN022866	MN022838	MN022810
GZRn110	Yuexiu	Male	MN022867	MN022839	MN022811
GZRn127	Haizhu	Male	MN022868	MN022840	MN022812
GZRn133	Haizhu	Female	MN022869	MN022841	MN022813
GZRn134	Haizhu	Male	MN022870	MN022842	MN022814
GZRn148	Liwan	Female	MN022871	MN022843	MN022815

Table S3 SEOV strains and their GenBank accession number for those sequences used to create L, M, and S segment phylogenetic trees

Segment	Strain	Accession number	Country	Collection date
**L**	Humber/2012	JX879770	United Kingdom	2012
	Cherwell/2013	KM948594.1	United Kingdom	2013
	ZT10/2007	EF581094.1	**Zhejiang province**	2003
	ZT71/2006	EF190551.1	**Zhejiang province**	2003
	Rn11-44/NGS/2011	MF149940.1	South Korea	2011
	Fj372/2013	KP645196.1	**Fujian province**	2013
	Hu02-258/NGS/2002	MF149951.1	South Korea	2002
	Rn10-134/NGS/2010	MF149938.1	South Korea	2010
	Rn10-145/NGS/2010	MF149939.1	South Korea	2010
	Rn11-53/NGS/2011	MF149941.1	South Korea	2011
	IR33/1984	KX079471.1	United Kingdom	1984
	IR162/1984	KX079474.1	United Kingdom	1984
	Rn84/2013	MG972931.1	USA	2013
	SEOV/NL/Rn2125/2016	MG764082.1	Netherlands	2016
	SEOV/NL/Rn2147/2016	MG764083.1	Netherlands	2016
	Northeast/Baltimore/2002	KT897724.1	USA	2002
	80_39/1991	X56492.1	South Korea	1991
	Tchoupitoulas/POR/1984	KU204958.2	USA	1984
	JiangxiXianjianRn-07/2011	KP900346.1	**Jiangxi province**	2011
	DPRK08/2011	JX853574.1	North Korea	2011
	IR473/1984	KX079468.1	United Kingdom	1984
	Seoul-DN2/2014	KX289952.1	**Heilongjiang province**	2014
	LYON/Rn/FRA/2013/LYO852/2011	KF387723.1	France	2011
	Humber/2012	KM948596.1	United Kingdom	2012
	Rn10-134/2010	MF149936.1	South Korea	2010
	Rn10-145/2010	MF149937.1	South Korea	2010
**M**	K24-e7/2000	AF288652.1	**Zhejiang province**	2000
	K24-v2/2000	AF288654.1	**Zhejiang province**	2000
	ZT10/2003	DQ159911.1	**Zhejiang province**	2005
	IR33/1984	KX079472.1	United Kingdom	1984
	IR162/1984	KX079475.1	United Kingdom	1984
	IR473/1984	KX079469.1	United Kingdom	1984
	Rn84/2013	MG972934.1	Netherlands	2013
	Rn10-134/NGS/2010	MF149942.1	South Korea	2010
	Rn10-145/NGS/2010	MF149943.1	South Korea	2010
	Rn11-44/NGS/2011	MF149944.1	South Korea	2011

	Rn11-53/NGS/2011	MF149945.1	South Korea	2011
	NL/Rn2147/2016	MG764081.1	Netherlands	2016
	NL/Rn2125/2016	MG764080.1	Netherlands	2016
	Fj372/2013	KP645197.1	**Fujian province**	2013
	Cherwell/2013	KM948593.1	United Kingdom	2013
	Humber/2012	JX879768.1	United Kingdom	2012
	Humber/2012	KM948597.1	United Kingdom	2012
	Northeast/Baltimore/2002	KT897725.1	USA	2002
	ZT71/2003	EF117248.1	**Zhejiang province**	2003
	80_39/1991	NC_005237.1	South Korea	1991
	Tchoupitoulas/POR/1984	KU204959.2	USA	1984
	HaixingRn40/2010	GU592924	**Zhejiang province**	2010
	RuianRn242/2010	GU592928.1	**Zhejiang province**	2010
	LongwanRn58/2010	GU592930.1	**Zhejiang province**	2010
	DPRK08/2011	JX853576.1	North Korea	2011
	HBQ17/2013	KM233655.1	**Hebei province**	2013
	Seoul/DN2/2014	KX289953.1	**Heilongjiang province**	2014
	Orthohantavirus/158446/2015	KY639551.1	**Shandong province**	2015
	Orthohantavirus/N23/2013	KY639555.1	**Shandong province**	2013
	JNDX70/2014	KY639558.1	**Shandong province**	2014
	JX20141159/2014	KY639563.1	**Shandong province**	2014
	JX20131067/2013	KY639569.1	**Shandong province**	2013
	JX20140544/2014	KY639572.1	**Shandong province**	2014
	JX20141138/2014	KY639573.1	**Shandong province**	2014
	LZSF8/2014	KY639580.1	**Shandong province**	2014
	2015B21/2015	KY639585.1	**Shandong province**	2015
	YY20151056/2015	KY639588.1	**Shandong province**	2015
	2015E5/2015	KY639601.1	**Shandong province**	2015
	YY140512/2014	KY639613.1	**Shandong province**	2014
	YY140523/2014	KY639613.1	**Shandong province**	2014
	YY140532/2014	KY639618.1	**Shandong province**	2014
	Orthohantavirus/AQ150325/2015	KY639619.1	**Shandong province**	2015
	ZBLZ19/2014	KY639620.1	**Shandong province**	2014
	Hu02-258/NGS/2002	MF149946.1	South Korea	2002
**S**	GuangzhouRn36/2010	GU592948.1	**Guangdong province**	2010
	Seoul/CSG5/2007	AB618112.1	Vietnam	2007
	SEOV/NL/Rn2147/2016	MG764079.1	Netherlands	2016
	SEOV/NL/Rn2125/2016	MG764078.1	Netherlands	2016
	Hu02-258/NGS/2002	MF149954.1	South Korea	2002
	Rn11-53/NGS/2011	MF149950.1	South Korea	2011
	Rn11-44/NGS/2011	MF149949.1	South Korea	2011
	Rn10-145/NGS/2010	MF149948.1	South Korea	2010
	Rn10-134/NGS	MF149947.1	South Korea	2010
	DN2/2014	KX289954.1	**China**	2014

Northeast/Baltimore/2002	KT897726.1	USA	2002
IR162/1984	KX079476.1	United Kingdom	1984
IR33/1984	KX079473.1	United Kingdom	1984
Fj372/2013	KP645198.1	**Fujian province**	2013
Humber/2012	JX879769.1	United Kingdom	2012
Humber/2012	KM948598.1	United Kingdom	2012
Cherwell/2013	KC626089.1	United Kingdom	2013
DPRK08/2011	JX853575.1	North Korea	2011
ZT10/2003	AY766368.1	**Zhejiang province**	2003
ZT71/2003	AY750171.1	**Zhejiang province**	2003
IR473/1984	KX079470.1	United Kingdom	1984
Rn84/2013	MG972939.1	Netherlands	2013
Tchoupitoulas/POR/1984	KU204960.2	USA	1984
80_39 /1991	NC_005236.1	South Korea	1991
Hantavirus/R22/2002	AF488707.1	**Henan province**	2002
Seoul-virus-2000	AY006465.1	**Heilongjiang province**	2000
Hantavirus-BjHD01/2004	AY627049.2	**Beijing**	2004
Hantavirus-JUN5-14/2004	DQ217791.1	**Shandong province**	2004
CixiRf23/2007	FJ803201.1	**Zhejiang province**	2007
CixiRn169/2007	FJ803207.1	**Zhejiang province**	2007
OuhaiRn189/2007	FJ803211.1	**Zhejiang province**	2007
RuianRn33/2007	FJ803215.1	**Zhejiang province**	2007
Singapore/06-RN41/2006	GQ274944.1	Singapore	2006
HuBJ22/2006	GQ279379.1	**Beijing**	2006
Rn-M11/2006	GQ279383.1	**Beijing**	2006
HuBJ15/2005	GQ279390.1	**Beijing**	2005
HebeiMm7/2010	GU592935.1	**Hebei province**	2010
ShenyangRn19/2010	GU592950.1	**Liaoning province**	2010
RuianRn180/2010	GU592953.1	**Zhejiang province**	2010
WuhanRn10/2009	JQ665920.1	**Hubei province**	2009
REPLONGES-Hu-FRA/2012/12-0882A/2012	KC902522.1	France	2012
Gongzhuling42/2009	KF745942.1	**Jilin province**	2009
Gongzhuling415/2011	KF745949.1	**Jilin province**	2011
Taonan52/2011	KF745950.1	**Jilin province**	2011
MANTENAY-MONTLIN/Rn/FRA/2015	KX064273.1	France	2015
JX20131073/2013	KY639669.1	**Shandong province**	2013
YY20140523/2014	KY639671.1	**Shandong province**	2014
ZBLZ34/2014	KY639679.1	**Shandong province**	2014
XJ5-2011/2011	KY807170.1	**Jiangxi province**	2011
Hu02-294-NGS/2002	MF149955.1	South Korea	2002

Note: Country in bold represent the areas that belong to China. There are total 11 provinces (Zhejiang, Fujian, Jiangxi, Heilongjiang, Hebei, Shandong, Guangdong, Henan, Liaoning, Hubei, and Jilin) and one independently administered municipal district (Beijing).

Table S4 Nucleotide length, best-fit model, and MCMC chain length for each gene segment of SEOV

Gene	Nucleotide length (bp)	Substitution models[1]	Relax-clock models[2]	Tree models	MCMC chain length[3]
L	6288	GTR+G+I	UCLD	Bayesian SkyGrid	200
M	3402	GTR+G+I	UCLD	Bayesian Skyline	100
S	903	GTR+G	UCLD	Bayesian Skyline	100

[1] The nucleotide substitution models were determined by jModeltest2.1.7.

[2] UCLD represents uncorrelated lognormal clock model.

[3] The unit was million.
